# Educational Differences in Cohort Fertility Across Sub-national Regions in Europe

**DOI:** 10.1007/s10680-020-09562-0

**Published:** 2020-08-10

**Authors:** Jessica Nisén, Sebastian Klüsener, Johan Dahlberg, Lars Dommermuth, Aiva Jasilioniene, Michaela Kreyenfeld, Trude Lappegård, Peng Li, Pekka Martikainen, Karel Neels, Bernhard Riederer, Saskia te Riele, Laura Szabó, Alessandra Trimarchi, Francisco Viciana, Ben Wilson, Mikko Myrskylä

**Affiliations:** 1 Max Planck Institute for Demographic Research, Rostock, Germany; 2 Federal Institute for Population Research, Wiesbaden, Germany; 3 Vytautas Magnus University, Kaunas, Lithuania; 4 Department of Sociology, Stockholm University Demography Unit (SUDA), Stockholm, Sweden; 5 Statistics Norway, Oslo, Norway; 6 Hertie School of Governance, Berlin, Germany; 7 Department of Sociology and Human Geography, University of Oslo, Oslo, Norway; 8 Population Research Unit, University of Helsinki, Helsinki, Finland; 9 University of Antwerp, Antwerp, Belgium; 10 University of Vienna, Vienna Institute of Demography, Austrian Academy of Sciences, Vienna, Austria; 11 Statistics Netherlands, The Hague, Netherlands; 12 Hungarian Demographic Research Institute, Budapest, Hungary; 13 Institut National D’études Démographiques (INED), Paris, France; 14 Institute of Statistics and Cartography of Andalusia, Sevilla, Spain; 15 London School of Economics and Political Science, London, UK

**Keywords:** Education, Sub-national region, Fertility rate, Cohort fertility, Empirical Bayesian, Europe

## Abstract

Educational differences in female cohort fertility vary strongly across high-income countries and over time, but knowledge about how educational fertility differentials play out at the sub-national regional level is limited. Examining these sub-national regional patterns might improve our understanding of national patterns, as regionally varying contextual conditions may affect fertility. This study provides for the first time for a large number of European countries a comprehensive account of educational differences in the cohort fertility rate (CFR) at the sub-national regional level. We harmonise data from population registers, censuses, and large-sample surveys for 15 countries to measure women’s completed fertility by educational level and region of residence at the end of the reproductive lifespan. In order to explore associations between educational differences in CFRs and levels of economic development, we link our data to regional GDP per capita. Empirical Bayesian estimation is used to reduce uncertainty in the regional fertility estimates. We document an overall negative gradient between the CFR and level of education, and notable regional variation in the gradient. The steepness of the gradient is inversely related to the economic development level. It is steepest in the least developed regions and close to zero in the most developed regions. This tendency is observed within countries as well as across all regions of all countries. Our findings underline the variability of educational gradients in women’s fertility, suggest that higher levels of development may be associated with less negative gradients, and call for more in-depth sub-national-level fertility analyses by education.

## Introduction

Research on variation in fertility in contemporary societies often focuses on the relationship between education and fertility (Gustafsson and Kalwij 2006; Kreyenfeld and Konietzka 2017; Sobotka et al. 2017). There is evidence that the typically negative relationship between women’s education and fertility has varied across place (Beaujouan et al. 2016; Klesment et al.^2014^; Van Bavel et al. 2018; Wood et al.^2014^) and time (Andersson et al. 2009; Jalovaara et al. 2019; Kravdal and Rindfuss 2008; Neels and De Wachter 2010). In a number of higher-income countries, the negative relationship has been diminishing in recent cohorts (e.g. Jalovaara et al. 2019). However, most previous analyses on this relationship have been conducted at the country level, while paying little attention to potential variation in this relationship across regions within countries. National patterns are, however, composites of sub-national regional patterns. As regionally varying contextual conditions may affect fertility outcomes (Basten et al.^2012^; de Beer and Deerenberg 2007; Kulu 2013), exploring this dimension might improve our understanding of observed national-level patterns in educational gradients in fertility (Snyder 2001). A perspective beyond the national level also has value in light of globalisation theories predicting that affluent, developed sub-national regions across countries will become more similar to each other over time, while regional differences in living conditions within countries will increase (Veltz 2014). An empirical sub-national regional approach is essential for finding out whether such tendencies, with potential relevance for regional variation in educational gradients in fertility, indeed exist.

From a macro-perspective, socio-economic development is among the central determinants of fertility levels (Bryant 2007; Lee 2003). In the past, countries with higher levels of socio-economic development tended to have lower fertility levels. However, among contemporary high-income countries, this long-standing negative relationship has reversed (Luci-Greulich and Thévenon 2013; Myrskylä et al.^2009^). A similar tendency is observed within European countries, as the association of fertility with the level of economic development across sub-national regions has become less negative or even positive over the last decades (Fox et al.^2019^). These shifts in the regional-level association of development and fertility might be related to national-level tendencies towards a weaker negative relationship between education and fertility. That fertility levels in highly developed sub-national regions seem to gain ground as compared to fertility levels in less developed regions might be related to the fact that the high-educated are particularly concentrated in highly developed sub-national regions (Eurostat 2019). Our investigation aims to make a novel contribution by analysing for the first time for a large number of European countries the educational patterning of cohort fertility rates (CFRs) at the sub-national regional level. We seek to bridge previous studies on the relationships between education and fertility and development and fertility by exploring whether regional variation in CFRs by educational attainment is systematically associated with regional variation in economic development.

Existing research on educational differences in cohort fertility shows that higher-educated women have had lower CFRs at least since the early twentieth century (Skirbekk 2008). However, in the cohorts born up to the mid-1940s, there was convergence towards a two-child family model across educational groups, which in some countries led to a decrease in the educational gradient in CFR (Van Bavel et al. 2018). Moreover, the gap in childlessness between low- and middle-educated women, which has been an important factor in CFR differentials, declined among the mid-twentieth-century cohorts (Beaujouan et al. 2016). Further deviations from the well-known pattern of a negative educational gradient in CFRs have been reported for female cohorts born between the 1940s and the mid-1970s in Northern and North-Western Europe, where gradients have narrowed, and are often no longer observable (Andersson et al. 2009; Jalovaara et al. 2019; Kravdal and Rindfuss 2008; Neels and De Wachter 2010). So far, however, there has been no overall convergence in cohort fertility among educational groups across high-income countries (Sobotka et al. 2018). The magnitude of fertility gradients continues to vary greatly across countries, with Central and Eastern European countries as well as German-speaking countries continuing to witness particularly strong gradients (Beaujouan et al. 2016; Klesment et al. 2014; Neyer and Hoem 2008; Wood et al. 2014). As far as we know, no previous study has explored variation in educational gradients in fertility at the sub-national level.

Women’s cohort fertility results from consecutive decisions and events in the life course that are shaped by contextual factors (Huinink and Kohli 2014; Thomson et al.^2013^). Thus, at the sub-national level, cohort fertility is subject to regional contextual conditions across the reproductive lifespan (Kulu 2013; Kulu, Vikat, and Andersson 2007). As we will discuss next, regionally varying contextual conditions might affect childbearing opportunity structures differently depending on women’s educational levels and can therefore lead to variation between regions in the educational gradient in fertility. Firstly, economically more developed regions tend to have higher regional living costs due to factors like expensive housing (Kurre 2003). While this is likely to depress childbearing by contributing to high direct costs of children (Dettling and Kearney 2014; Flynn 2017; Mulder 2013), the childbearing of high-educated women might be less sensitive to such mechanisms because they are more likely to have high household income levels due to assortative mating (Esping-Andersen 2009; Konietzka and Kreyenfeld 2010). Secondly, women’s employment has recently become an important prerequisite for childbearing in most European countries (Matysiak and Vignoli 2008), particularly among high-educated women (Kreyenfeld and Andersson 2014; Wood and Neels 2017). Regional employment prospects (Bujard and Scheller 2017; Kravdal 2002), which tend to be better in economically more developed regions (Dunford 1996), are particularly important for the high-educated women. However, there might also be counteracting mechanisms, as in developed regions, labour markets may be more competitive, which could depress fertility, particularly among the high-educated (Kulu 2013; Kulu and Washbrook 2014).

Thirdly, the availability of flexible working arrangements that support work–family reconciliation, such as working remotely from home, is likely to be better in more developed regions (Fox et al. 2019) and might encourage fertility of high-educated employees who often face reconciliation challenges (Golden 2001). Fourthly, the regional availability of childcare services seems to have particularly strong positive effects on the fertility of high-educated women (Rindfuss et al.^2010^), most likely because of the high opportunity costs they face when having children (Wood et al. 2017). It is plausible that higher concentrations of the high-educated (Eurostat 2019) and dual-earner couples (de Meester and Van Ham 2009) in more developed regions contribute to more demand for and therefore better availability of childcare services in these regions, which could in turn lead to a less negative educational gradient.

To sum, it appears that higher living costs, better employment opportunities, and better access to flexible work arrangements and childcare services, which all seem positively associated with the economic development level of a region (Dunford 1996; Fox et al. 2019; Kosfeld et al. 2007; Kurre 2003), can overall enhance fertility of the higher-educated as compared to the less-educated women. Hence, these factors may contribute to a less negative educational gradient in fertility in more developed regions. Additionally, also normative and cultural factors (Kulu 2013; Mulder 2013), which are not discussed here, might importantly contribute to regional variation in fertility by educational attainment.

This study aims to describe the educational gradient of the female cohort fertility rate (CFR) at the regional level in contemporary Europe. We assess (1) whether there is sub-national regional variation in the educational gradient in CFRs, and (2) whether this variation is systematic by regional level of development. Based on our theoretical considerations, we expect to find that negative educational fertility gradients are more common among women living in less economically developed regions, and that these gradients might be low or even positive in more developed regions. In our analyses, we investigate the cohort fertility of women born in the late 1960s and early 1970s in 15 European countries. These cohorts are of interest at least for two reasons. First, they provide an up-to-date cohort perspective to fertility in Europe, i.e. they recently completed their childbearing. Second, for them, we witness in some European countries substantial shifts towards less negative or even slightly positive gradients in cohort fertility by educational attainment. For these women, we are able to measure number of children, level of education, and region of residence at the end of the reproductive career.

Women may move across regions over their life course—before, during, and after having (any) children. While sub-national migration and family formation are often closely interrelated life-course steps, long-distance moves are less common at higher parities and higher reproductive ages (Dommermuth and Klüsener 2019; Kulu 2008; Michielin 2004). The higher-educated have a greater propensity to migrate, and their reasons for migration are often related to further education and employment. Thus, they typically move to economically developed regions and cities (Berry and Glaeser 2005). This pattern likely contributes to higher concentrations of the high-educated in the more developed regions.^1^ For the low-educated, family-related reasons, such as proximity to kin, are more relevant for moving decisions. Thus, their moves seem to be less dependent on the economic development level of the destination region (Dawkins 2006; Thomas 2019). Taken together, a woman’s region of residence at the end of her reproductive life, as measured in this study, may differ from the region where she lived during her prime childbearing years, possibly even in systematic ways. This can bias attempts to investigate associations between regional contextual factors and our measured fertility outcomes. This study thus does not aim at validating a causal link between contextual factors and fertility. Instead, it attempts to identify, based on the region of residence of women at the end of the reproductive lifespan, regularities in the regional-level relationship between education and cohort fertility.

## Data and Methods

The study is based on register, census, and large-scale survey data (see Table 1 for details). We cover 15 countries: Austria, Belarus, Belgium, Finland, France, Germany, Greece, Hungary, Ireland, Lithuania, the Netherlands, Norway, Romania, Spain, and Sweden. The analyses focus on native-born women born between 1964 and 1970. Cohort fertility, highest educational attainment, region of residence, and level of economic development are measured at the end of the reproductive career. In most countries, the data reflect the achieved fertility as of 2011. All women were aged 40 or older at the time of the measurement.

**Table 1:** Data sources of the study in 15 European countries

Country	Cohorts	Sample (%)	Data type	Measurement date	Age at measurement
Austria	1965–1970	> 1	Microcensus + survey	2012–2013/2016^a^	42–46
Belarus	1965–1968	10	Census	14. –24.10.2009	41–45
Belgium	1964–1966	100	Register	31.12.2006^b^	40–42
Finland	1966–1970	10	Register	31.12.2012^c^	42–46
France	1965–1970	1	Survey	26.2.2011	40–45
Germany	1964–1970	1	Microcensus	2008/2012/2016^d^	41–48
Greece	1965–1970	10	Census	10. –20.5.2011	40–46
Hungary	1966–1970	100	Census	1.10.2011	41–45
Ireland	1965–1970	10	Census	10.4.2011	41–45
Lithuania	1966–1970	100	Census	1.3.2011	41–45
The Netherlands	1966–1970	100	Register	31.12.2011	41–43
Norway	1966–1970	100	Register	31.12.2011	41–45
Romania	1965–1970	10	Census	20. –31.10.2011	40–45
Spain	1966–1970	9	Census	11.1.2011	41–45
Sweden	1966–1970	100	Register	31.12.2012	40–44

^a^Data sources in Austria are microcensuses in 2012 (4th quarter) and 2016 (4th quarter), Austrian Gender and Generations Survey gathered from September 2012 to March 2013, and Basic Social Science Research for Vienna Survey gathered from October 2012 to July 2013

^b^In Belgium, education is measured in the census conducted on 1 October 2001 when women were aged 34 to 37

^c^In Finland, education and region were measured on 31 December 2007 when women were aged 37 to 41

^d^Data sources for Germany are microcensuses in 2008, 2012, and 2016 gathered throughout the year

The regional classification is based on the Nomenclature of Territorial Units for Statistics (NUTS) classification by Eurostat (2011), a sub-regional categorisation of territorial units in the European Union. For most countries, we use the NUTS 2 level of classification, which covers regions and smaller countries with between 800,000 and three million inhabitants. “Appendix 1” provides detailed information on the regional categorisation.

In register data, information on the region of residence is derived from registers on the place of dwelling. In survey data, it is self-reported. In census data, it is either self-reported or obtained from registers and corrected, where necessary, based on self-reports. We measure regional development using GDP (purchasing power standardised gross domestic product per capita) in 2011 extracted from the Eurostat database (Eurostat 2018). We also considered other development measures, such as employee compensation, which focuses on household income. But as employee compensation is highly correlated with GDP across European regions and is not available for all regions in our dataset, we decided to use GDP.^2^ See “Appendix 2” for GDP per capita across regions.

The measurement of education is based on registers in the register data and self-reports in other data. We distinguish between low, medium, and high educational attainment following the International Standard Classification of Education (ISCED) (UNESCO 1999). High refers to education at the tertiary level (ISCED 1997 levels 5–6), including short-cycle tertiary level education. Medium refers to education at the higher secondary or post-secondary non-tertiary level (ISCED 1997 levels 3–4). Low refers to education at the lower secondary level or lower (ISCED 1997 levels 1–2).^3^ In Belarus, Greece, Ireland, and Romania, the classification is based on the standards used by IPUMS international (IPUMS 2018).^4^ See “Appendix 2” for the distribution of the educational attainment by region.

Fertility is measured as the mean total number of children per woman, corresponding to the cohort fertility rate. This number includes all children women have ever given birth to and is derived from self-reports in census or survey data and information on registered births in register data. In France, the Netherlands, and Norway, children given for adoption are linked to their adoptive parents instead of their biological parents. The country-specific mean age at the measurement of fertility was at least 42 in all cases except Belgium, where it was 41. Thus, completed fertility is particularly in Belgium slightly underestimated. Prior research indicates, however, that changes in the educational gradient of women are very small past this age (Andersson et al. 2009; Berrington et al.^2015^). In census-based data, women reporting unknown parity may cause errors in the parity estimates (Sobotka 2017). Among the countries in this study for which census or survey data are used, the small numbers of women with unknown parity are redistributed in Belarus, Germany, and Lithuania.

We use two types of linear regression models to analyse the association between CFR and women’s education. The first approach is a simple linear regression model that pools data across all countries and regions and has as the outcome fertility difference between two educational groups (for example, high versus medium education) and as covariate log of GDP at the regional level. This regression describes how the educational gradient in fertility varies across levels of GDP across all countries and regions. The second approach is a regression model that has the same outcome and covariate, but in addition country fixed effects. This model effectively estimates for each country the within-country association between the educational difference in fertility and log of GDP and provides an average of these within-country associations. The results from this model describe how, on average, the educational gradient in fertility relates to log of GDP within countries.

Our data include both regions with large and with small numbers of observations: in 9 out of the 15 countries, full population data are not available (see Table 1). One option to analyse these data would be to use raw, unadjusted CFRs as observed in the data. This approach has the disadvantage that our results might partially reflect more random small-sample variation than true heterogeneity. Therefore, we use an alternative but standard method of small area estimation to smooth out small-sample variation: the empirical Bayesian (EB) estimation (Assunção et al.^2005^; Longford 1999; Rao 2014). In this method, statistical power is borrowed from other educational groups and regions to limit noise in the fertility rate estimates. We assume that the number of children follows a Poisson distribution, and borrow strength for each educational group (1) from other educational groups within the region, (2) from the same educational groups in other regions within the country, and (3) from regularities in education-specific fertility schedules within the country. Regions with a small number of observations are influenced more by this procedure than regions with a large number of observations, and power is borrowed not only proportional to the size of the region but also to the GDP, such that regions similar in GDP borrow more strength from each other than regions that have different levels of GDP. “Appendix 3” shows details of the method. An important robustness check is to compare the CFRs as observed and after the EB adjustment. Appendices 4–6 show this comparison. Our main conclusions are based on the regression models, and we have analysed the sensitivity of our regression models to the EB adjustment. Appendices 9–11 show our main results based on unadjusted CFRs. These findings are stronger than those based on EB adjustment (reported in Results section). We consider that the more conservative EB-based results are more likely to reflect reality than the unadjusted measures.

## Results

### A Comparison at the Country Level

We first situate the sub-national analysis within the broader cross-country context in Europe. National CFRs independent of education range from 1.50 in Germany to 2.09 in Ireland (Table 2). Turning to the CFRs by education, it is relevant to note that the cross-country average of the share of women in each educational category is 32% for tertiary (range 16–53%), 53% for medium (36–73%), and 16% for low (2–29%) (“Appendix 2”). The medium-educated are the largest group in all but two countries (Finland and Norway), while the low-educated are the smallest group in all but one country (Greece). The educational gradient in CFRs is negative in almost all countries, but the magnitude of the gradient varies across countries and educational comparisons. Notably, high- and medium-educated women are, on average, more similar in their CFRs than medium- and low-educated women. The high-educated have, on average, fewer children than the medium-educated in all but one country, with the difference ranging from − 0.42 in Romania, to almost zero in Norway and Sweden, to 0.10 in Belgium. The medium-educated have fewer children than the low-educated, with the difference ranging from close to zero in Finland and Norway to − 0.68 in Romania and − 0.59 in Hungary. In all countries, our derived rates for the high-educated are below those for the low-educated, with the difference ranging from − 0.03 (Norway) and − 0.01 (Belgium) to − 0.69 (Hungary) and − 1.10 (Romania).

**Table 2:** Cohort fertility rate of women by level of education in 15 European countries

Country	High	Medium	Low	Total	Δ High–medium	Δ Medium–low	Δ High–low
Austria	1.38	1.62	1.98	1.61	− 0.19	− 0.33	− 0.52
Belarus	1.43	1.76	1.96	1.68	− 0.29	− 0.14	− 0.44
Belgium	1.74	1.65	1.78	1.72	0.10	− 0.11	− 0.01
Finland	1.81	1.99	1.97	1.90	− 0.15	− 0.01	− 0.16
France	1.76	1.87	2.10	1.87	− 0.09	− 0.22	− 0.31
Germany	1.40	1.51	1.67	1.50	− 0.11	− 0.16	− 0.27
Greece	1.54	1.69	2.09	1.76	− 0.14	− 0.33	− 0.48
Hungary	1.66	1.77	2.42	1.86	− 0.10	− 0.59	− 0.69
Ireland	1.88	2.10	2.38	2.09	− 0.22	− 0.28	− 0.50
Lithuania	1.56	1.90	2.06	1.80	− 0.29	− 0.14	− 0.43
The Netherlands	1.71	1.82	1.89	1.81	− 0.10	− 0.03	− 0.12
Norway	1.99	2.04	2.05	2.02	− 0.01	− 0.02	− 0.03
Romania	1.12	1.57	2.28	1.65	− 0.42	− 0.68	− 1.10
Spain	1.34	1.48	1.71	1.46	− 0.14	− 0.14	− 0.28
Sweden	1.93	1.94	2.04	1.94	0.00	− 0.10	− 0.10
Mean	1.62	1.78	2.03	1.78	− 0.14	− 0.22	− 0.36

### Variation Between and Within Countries by GDP

**Fig. 1:**
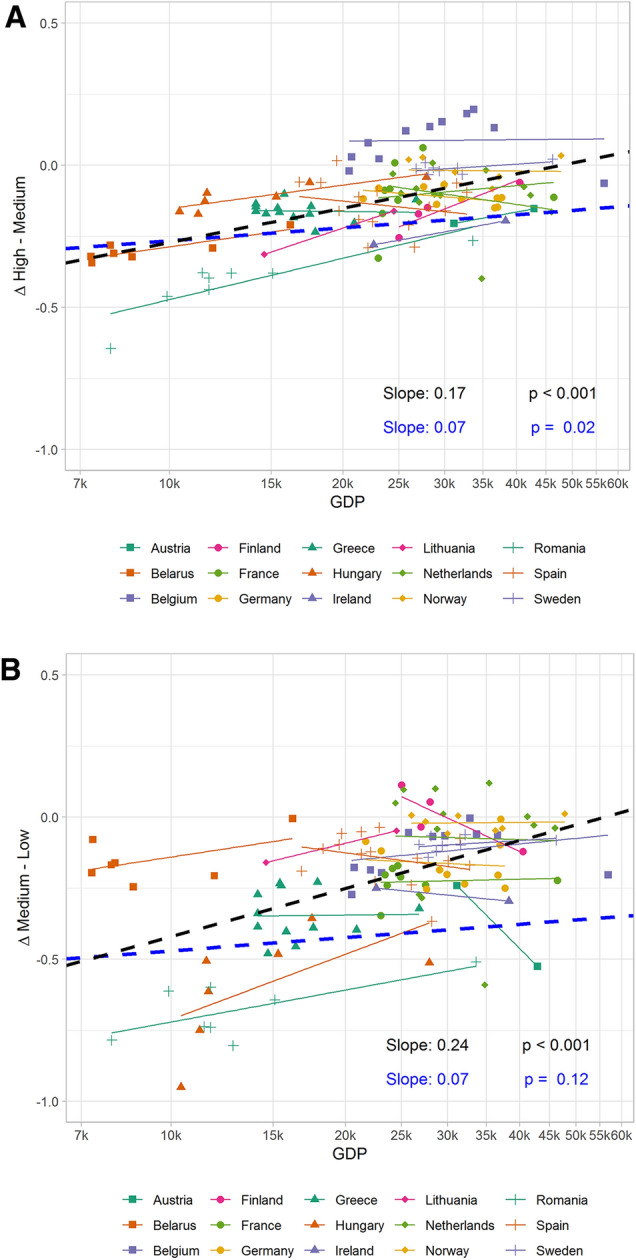

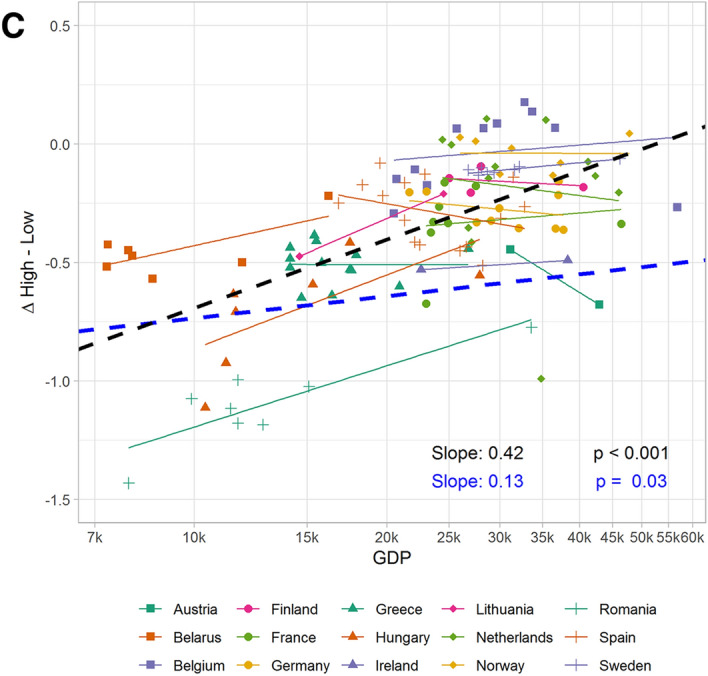
**a** Difference in cohort fertility rate between high- and medium-educated women according to the GDP per capita level of the region in 15 European countries. Regression lines are fitted for the global trend without (black dashed line) and with (blue dashed line) country fixed effects, and for the within-country trends for each country separately (solid lines). **b** Difference in cohort fertility rate between medium- and low-educated women according to the GDP per capita level of the region in 15 European countries. Regression lines are fitted for the global trend without (black dashed line) and with (blue dashed line) country fixed effects, and for the within-country trends for each country separately (solid lines). **c** Difference in cohort fertility rate between high- and low-educated women according to the GDP per capita level of the region of the 15 European countries. Regression lines are fitted for the global trend without (black dashed line) and with (blue dashed line) country fixed effects, and for the within-country trends for each country separately (solid lines)

Figure 1 plots for our complete set of regions the CFR difference between the high- and medium-educated (Fig. 1a), the medium- and low-educated (Fig. 1b), and the high- and low-educated (Fig. 1c) by log-transformed GDP.^5^ The overall pattern that emerges is that of a negative educational gradient that declines as the level of regional economic development increases, and that has considerable variation at any level of GDP. The regions with low GDP tend to display larger educational differences in CFRs. In a number of regions, the difference is reversed, i.e. the high-educated have a higher CFR than the low-educated, particularly among the more developed regions. See “Appendix 7” for CFR by education for all regions. Fertility differentials between educational groups tend to become smaller as we move from regions with lower GDP to regions with higher GDP. This is observed both within countries and across all regions of all countries. The regression lines superimposed on the graphs show a strong and significant correlation between higher levels of economic development and decreasing differences in fertility between educational groups for all three educational comparisons.

Whether this pattern is attributable to variation between or within countries can be tested by regressing the educational fertility difference on GDP while controlling for country fixed effects. The coefficients of these regressions are shown in the bottom-right corner of each of the figures. In each instance, the coefficients suggest that within countries, educational differences in fertility also tend to be smaller as the level of economic development of a region increases. The evidence for the within-country pattern is stronger in the high–medium comparison than in the medium–low comparison. As the figures illustrate, there are exceptions to the general pattern of a smaller gradient, as in some countries, the educational fertility gradient is not associated or positively associated with the level of GDP of a region. However, averaged across all countries, the evidence suggests that within countries, a higher level of economic development is also associated with smaller differences in fertility between educational groups. Additional analyses show that countries in Eastern and Central Europe strongly contribute to the within-country pattern. In a supplemental analysis that excludes Central and Eastern European countries, the cross-country association between the educational gradient and GDP persists, but the within-country association becomes flat (“Appendix 8”).

### Regions with the Highest GDP and Other Regions

**Fig. 2:**
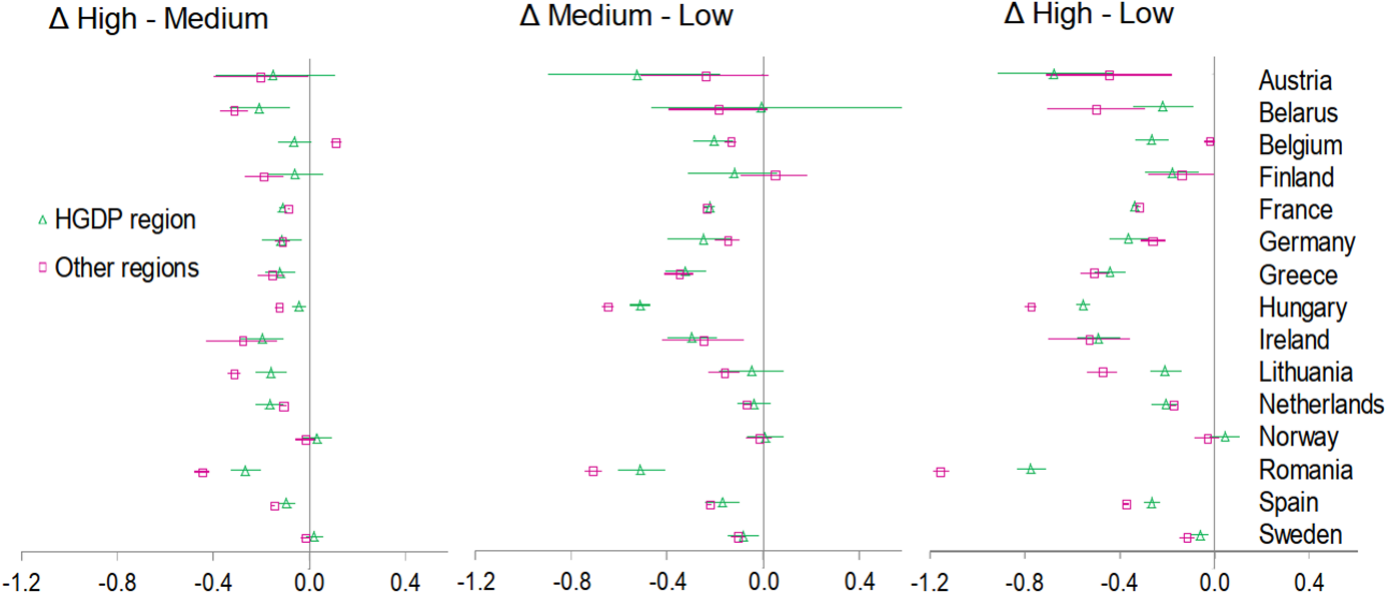
Difference in cohort fertility rate between two educational groups of women by country: the region with the highest GDP per capita value (HGDP region) and other regions of a country in 15 European countries. Figures also display 95% credible intervals of the point estimates with horizontal lines

In order to better understand within-country patterns, we additionally analyse educational gradients by comparing the economically most developed region to all other regions within each country (Fig. 2). There is a general tendency towards smaller educational differences in the most developed regions within countries: in all educational group comparisons, the educational difference averaged across countries is smaller in the region with the highest GDP. However, there is large variation across countries around this average tendency. The three panels shown in Fig. 2 indicate that the differences in the magnitudes of the educational gradient between the highest GDP region and other regions are particularly large in Eastern European countries, where the country-level magnitudes of the gradient are also large. In Norway, Sweden, Spain, and Greece, the differences are also smaller in the highest GDP region, but the differences relative to other regions are not as large. In Finland, France, the Netherlands, and Ireland, the differences between the highest GDP region and other regions are small; and in Belgium, Austria, and Germany, there are indications that the educational gradient is larger in the highest GDP regions than in other regions.

## Conclusion

Previous studies have shown that educational differences in women’s completed fertility vary between countries and over time. We show that educational gradients also vary across sub-national regions within countries in Europe, and that this variation is notable and quite systematic. Women educated to high and medium levels are, on average, more similar in their completed fertility than women educated to medium and low levels, and the gradient between the high- and low-educated in completed fertility narrows with increasing levels of economic development between and within countries. However, the variation between countries in the within-country pattern is noteworthy. For example, in Hungary, high-educated women have only 0.04 fewer children than medium-educated women in the most developed region, compared to 0.13 fewer children in other areas. Meanwhile, in Belgium, high-educated women have 0.06 fewer children than medium-educated women in the most developed region, but 0.11 more children in other areas.

We expected to find weaker negative gradients in cohort fertility in the more developed regions based on our theoretical considerations that the contextual conditions in such regions could lead to more similar childbearing patterns among women in different educational groups. For example, the fertility of the low-educated might be particularly depressed in these regions due to higher living costs, while the high-educated might benefit more from better access to employment, childcare services, and flexible work. In line with our expectations, we find that well-developed regions have smaller differentials in fertility by education. In this descriptive analysis, however, we were not able to test the importance of the discussed mechanisms. Further studies could validate the role of regional contextual factors for educational gradients in fertility by linking individual-level data to such factors at the time when childbearing decisions are made (see Hank 2002; Kulu 2013). The role of sub-national migration over the life course for the educational patterning of cohort fertility at the regional level also requires investigation. Given the sequential nature of childbearing and the evidence that cohort fertility masks parity-specific variation (Zeman et al. 2018), parity-specific analysis (see Fiori et al. 2014; Kulu and Washbrook 2014) may help to disentangle the mechanisms behind the observed patterns.

The environment women born in the late 1960s experienced during their prime childbearing years differed substantially across countries. An elaborated analysis of such between-country differences is beyond our focus, but we note that women in the former communist countries—Belarus, former East Germany (classified here as part of Germany^6^), Hungary, Lithuania, and Romania—experienced a very particular childbearing context (Billingsley 2010; Sobotka 2011). The female cohorts of the late 1960s were in their early twenties at the onset of the crisis of the Soviet Union in 1989. By then, many of those who had not entered university had already become mothers, while many of those who were students finished their education after the onset of the crisis and were thus more likely to postpone childbearing. These circumstances contributed to strong variation in fertility in the cohorts studied (Kreyenfeld 2006). The timing of the crisis may have also contributed to some of the strong regional patterns we observe in the Central and Eastern European countries. Moreover, the high levels of regional inequality in these countries (Petrakos 2001) may have further contributed to regional variation in educational gradients.

Our data sources vary by country. Measurement is likely to be more accurate in register than in census or survey data. Quality assessment of the small-sample data sources used in the study in Austria (Neuwirth 2015; Statistics Austria 2018; Verwiebe et al. 2014), France (INSEE 2013, 2014), and Germany (Federal Statistical Office and Statistical Offices of the Federal States 2018) showed relatively high overall response rates (78–95%), but lower rates among the low-educated and varying rates by region, with the rates being lower in capital regions. We cannot rule out the possibility that measurement error affected the results of this study, but it is unlikely that it would have led to the main results, because the error would need to be differently selective by educational attainment across regions. We were also unable to assess the sensitivity of our results to regional categorisation (“modifiable areal unit problem”) (Openshaw 1984).

This study underlines the variability of the educational gradient in fertility and shows that a sub-national regional approach can advance our understanding of the dynamics of educational differentials in fertility. We document an overall negative gradient between cohort fertility and level of education, and notable variation in the magnitude of the gradient across sub-national regions. While weaker negative gradients are generally found in more economically developed regions in contrast to less developed regions, notable differences can be observed in the within-country patterning of the gradient. The high fertility of high-educated women relative to medium- (or low-) educated women in more developed regions suggests that the overall negative educational gradients in cohort fertility at the country level are more strongly driven by women living in less economically prosperous sub-national regions. Such tendency is most clearly evident in Central and Eastern Europe, where country-level negative educational gradients in cohort fertility remain strong. Given that the shares of women with high educational attainment are particularly large in well-developed regions, these findings may help to explain why overall fertility has been relatively high in well-developed regions in Europe in recent years. A longitudinal approach covering successive birth cohorts and information on place of residence over the life course would be useful to complement these descriptive findings.

Additional material: an interactive map showing women’s cohort fertility rate by level of education at the sub-national regional level in 15 European countries: https://fertility.shinyapps.io/cfr_edu_region/; the cohort fertility rates in digital format and the code used to generate the interactive map: https://github.com/DemogrFertility/cfr_edu_region.

## Appendix 1: Description of the Sub-national Regional Categorisation in 15 European Countries

The NUTS categorisation is strongly linked to existing administrative divisions in a country, and also considers the general character and population size of the region. This categorisation has three levels, and we generally aimed to use the NUTS 2 level, which covers regions with between 800,000 and three million inhabitants. At the NUTS 1 level, on the other hand, many smaller countries would consist of one region only. NUTS 2-level data were analysed for Belarus, Belgium, Finland, Greece, Hungary, Ireland, the Netherlands, Norway, Romania, Spain, and Sweden. These regions generally have populations between 800,000 and three million inhabitants. In Austria, France, and Germany, limited sample sizes forced us to conduct the analysis at a higher level of geographic detail. In Austria, the capital city of Vienna (NUTS 2) was compared with the rest of the country. For France, we excluded overseas territories, and used the NUTS 1 level. For Germany, a modified version of the NUTS 1 level was used to compensate for the small numbers in some regions due to the sample used. For Finland, we excluded the Åland islands; and for Spain, we excluded the Canary Islands and the Balearic Islands, Ceuta and Melilia, due to their small population sizes and distinct cultures. For Lithuania, which back in 2011 consisted of just one NUTS 2 region, we separated out the capital city of Vilnius (a LAU1 level unit in the classification of Eurostat) from the rest of Lithuania. Belarus is not an EU country, but a corresponding classification has been developed for it; please see http://riate.cnrs.fr/wp-content/uploads/2015/03/M4D_20121220_TR_russia.pdf.

## Appendix 2: Descriptive Characteristics of the Study Population by Sub-national Region in 15 European Countries

Note: Results for Austria, France, and Spain are shown as weighted. Sub-national regions within a country are ranked by the GDP per capita of the region, from highest to lowest. Population-weighted average GDP per capita was used for regions containing more than one NUTS region. In Lithuania, the GDP per capita value was not available for the chosen regional classification, and the value for Vilnius county, a larger area that also covers the surrounding areas of Vilnius city (NUTS 3 level region), had to be used as a proximate estimate. The total rows show for the GDP per capita the non-weighted country averages of the regional values.

## Appendix 3: Empirical Bayesian Estimation

We use a vector-based empirical Bayesian approach to estimate region- and education-specific cohort fertility rates and their credible intervals (Assunção et al. 2005; Longford 1999). The vector-based approach borrows strength not only from other regions in the same country based on the sample sizes of these regions and their similarity to the GDP of the region in question, but from other educational groups in the same region and from regularities in educational fertility schedules across regions. The estimation method can be described as follows. Suppose the total number of women from selected cohorts observed from country $c \left( {c = 1, \ldots ,C} \right)$, region $r \left( {r = 1, \ldots ,R_{c} } \right)$ with education level $e \left( {e = 1,2,3} \right)$ is denoted as $NWomen_{c,r,e}$ and the number of children is denoted by $NChild_{c,r,e}$. The crude cohort fertility rate is denoted by $\hat{\lambda }_{c,r,e} = NChild_{c,r,e} /NWomen_{c,r,e}$ and $\hat{\lambda }_{c,r} = NChild_{c,r} /NWomen_{c,r}$, where

$$ NWomen_{c,r} = \left( {NWomen_{c,r,1} , NWomen_{c,r,2} , NWomen_{c,r,3} } \right), $$

and

$$ NChild_{c,r} = \left( {NChild_{c,r,1} , NChild_{c,r,2} , NChild_{c,r,3} } \right). $$

Suppose the *real* cohort fertility rate $\lambda_{c,r,e}$ follows:

$$ NChild_{c,r,e} |\lambda_{c,r,e} \sim Poisson\left( {NWomen_{c,r,e} \times \lambda_{c,r,e} } \right) $$

The distance in GDP between region $r_{1}$ of country $c_{1}$ and region $r_{2}$ of country $c_{2}$ is defined as

$$ d_{{c_{1} ,c_{2} ;r_{1} ,r_{2} }} = \left\{ {\begin{array}{*{20}l} {|GDP_{{c_{1} ,r_{1} }} - GDP_{{c_{2} ,r_{2} }} |/range_{{1 \le c \le C,1 \le r \le R_{c} }} \left( {GDP_{c,r} } \right)} \hfill & {if\;c_{1} = c_{2} } \hfill \\ 1 \hfill & {if\;c_{1} \ne c_{2} } \hfill \\ \end{array} } \right. $$

The vectorial regional shrinkage estimator for the cohort fertility rate is denoted as:

$$ \lambda_{c,r}^{*} = \hat{\lambda }_{c,r} + \tau_{c,r} \left( {E\left( {\lambda_{c,r} } \right) - \hat{\lambda }_{c,r} } \right), $$

where $E\left( {\lambda_{c,r} } \right)$ is the cohort fertility rate of region $r$ and $\tau_{c,r}$ shrinking factor. $E\left( {\lambda_{c,r} } \right)$ is estimated as $\hat{E}\left( {\lambda_{c,r} } \right) = \sum\nolimits_{{r_{i} = 1}}^{Rc} {\left[ {\left( {1 - d_{{c,c;r,r_{i} }} } \right) \times \hat{\lambda }_{{c,r_{2} }} } \right]}$ by borrowing information from other regions in country $c$ according to their distances in GDP. The vectorial shrinkage estimator $\lambda_{c,r}^{*}$ shrinks a vector of regional cohort fertility rate estimates towards a more typical pattern of regional fertility estimates within a country, with more shrinkage when the distance in GDP is smaller. The shrinking factor $\tau_{c,r}$ is estimated using moments estimation, as proposed by Assunção et al. 2005, which gives larger values (i.e., more shrinkage) when the sampling noise of a regional estimate is expected to be large relative to the variability of the estimates across regions within a country. It follows that shrinkage is larger for regions with smaller sample sizes. Sensitivity analysis showed that the results were robust when different distance matrixes were defined based on the GDP. All CIs (credible intervals) were estimated based on 10,000 bootstrapping replications. The analysis was performed using R version 3.4.1.

## Appendix 7

Cohort fertility rate of women by educational attainment and sub-national region in 15 European countries. Empirical Bayesian cohort fertility rates.

Note: Results for Austria, France, and Spain are based on a weighted sample. Sub-national regions within a country are ranked by the GDP (per capita) of the region, from highest to lowest. 95% CI = 95 percent credible interval. Meckl.-West. Pom. and Sax.-Anh. = Mecklenburg-Western Pomerania and Saxony-Anhalt.

## Appendix 8

Coefficients and *p* values from linear models regressing differences in cohort fertility rates between educational groups and educational group-specific cohort fertility rates on logged GDP per capita: without and with country fixed effects in the sample of 15 European countries. Estimation is based on Empirical Bayesian cohort fertility rates.

Coefficients and *p* values from linear models regressing differences in cohort fertility rates between educational groups and educational group-specific cohort fertility rates on logged GDP per capita: without and with country fixed effects in the sample of 15 European countries. Estimation is based on observed cohort fertility rates.

## Appendix 9

The difference in cohort fertility rate between high- and medium-educated women according to the GDP per capita level of the region of 15 European countries. Regression lines are fitted for the global trend without (black dashed line) and with (blue dashed line) country fixed effects, and for the within-country trends for each country separately (solid lines). Estimation is based on observed cohort fertility rates.

## Appendix 10

The difference in cohort fertility rate between medium- and low-educated women according to the GDP per capita level of the region of 15 European countries. Regression lines are fitted for the global trend without (black dashed line) and with (blue dashed line) country fixed effects, and for the within-country trends for each country separately (solid lines). Estimation is based on observed cohort fertility rates.

## Appendix 11

The difference in cohort fertility rate between high- and low-educated women according to the GDP per capita level of the region of 15 European countries. Regression lines are fitted for the global trend without (black dashed line) and with (blue dashed line) country fixed effects, and for the within-country trends for each country separately (solid lines). Estimation is based on observed cohort fertility rates.

## Footnotes

1 Across Europe in 2018, 49% of women aged 30–34 had tertiary education and 15% had primary education, while in the capital regions, the respective shares were 58% and 13% (Eurostat 2019). In the cohorts under study, the educational levels were generally lower.

2 Based on own calculations of the European regional database (Cambridge Econometrics 2018), the correlation between GDP per capita and employee compensation was 0.97 across NUTS 2 levels in 2011.

3 In the Nordic countries, the small shares of women with missing information on educational attainment are classified as low-educated (< 3% in Norway, < 1% in Sweden and Finland).

4 In order to follow the ISCED as closely as possible, we classify technical education college degrees (Greece), third-level non-degree qualifications (Ireland), and short-term post-secondary (associate) degrees (Romania) as tertiary (see also OECD 2015).

5 See “Appendix 2” for within-country variation in educational attainment. In all but four countries (Belgium, Germany, the Netherlands, Spain), the share of high-educated women was largest in the region with the highest GDP.

6 The following regions belonged to the German Democratic Republic until 1990: Berlin, Brandenburg, Mecklenburg-Western Pomerania, Saxony, Saxony-Anhalt, and Thuringia.
